# Mutations in the MTHFR gene are not associated with Methotrexate intolerance in patients with juvenile idiopathic arthritis

**DOI:** 10.1186/s12969-016-0071-y

**Published:** 2016-02-29

**Authors:** Andrea Scheuern, Nadine Fischer, Joseph McDonald, Hermine I. Brunner, Johannes-Peter Haas, Boris Hügle

**Affiliations:** German Center for Pediatric and Adolescent Rheumatology (DZKJR), Gehfeldstrasse 24, 82467 Garmisch-Partenkirchen, Germany; Department of Rheumatology, Cincinnati Children’s Hospital Medical Center, Cincinnati, OH USA

**Keywords:** Juvenile idiopathic arthritis, Methotrexate Intolerance, MTHFR, Classical Conditioning, Methotrexate Toxicity

## Abstract

**Background:**

Methotrexate (MTX) intolerance is a frequent problem of long-term treatment in juvenile idiopathic arthritis (JIA). Mutations in the methylentetrahydrofolate reductase (MTHFR) gene may increase toxicity of MTX, potentially constituting an initial stimulus for this conditioned response. The objective of this study was to investigate the relationship of common MTHFR gene mutations and occurrence of MTX intolerance in pediatric patients with JIA treated with MTX.

**Methods:**

Consecutive JIA patients on at least 3 months of MTX treatment were included in this study. Intolerance to MTX was determined using the Methotrexate Intolerance Severity Score (MISS) questionnaire, and MTX intolerance was defined as MISS values of ≥ 6. Presence of the two most common mutations in the MTHFR gene (C677T and A1298C) was tested using a PCR assay. Results were analyzed using descriptive and non-parametric statistics.

**Results:**

196 patients were included (73 % female). Of those, 93 (46 %) showed MTX intolerance. 168 patients were genotyped for C677T and A1298C. MTX intolerance was not found to be significantly more frequent among patients with hetero- and homozygous or homozygous mutations C677T or A1298C compared to wild type or heterozygous mutations. Analysis of the correlation between numbers of mutations in these two loci to the MISS score did not yield a statistically significant correlation.

**Conclusion:**

Mutations in the MTHFR gene were not found to be significantly more frequent in JIA patients intolerant to MTX. Toxicity associated with the MTHFR gene seems to result from mechanisms different to those involved in clinical MTX intolerance.

**Electronic supplementary material:**

The online version of this article (doi:10.1186/s12969-016-0071-y) contains supplementary material, which is available to authorized users.

## Introduction

Methotrexate (MTX) has become the mainstay of treatment of polyarticular JIA in the course of the last 25 years [1]. Low-dose treatment with MTX shows few serious side effects, limited mostly to transient hepatotoxicity and bone marrow suppression in JIA [2, 3]. The most common side effects leading to MTX discontinuation in clinical practice are gastrointestinal symptoms including intolerance in the form of aversion, nausea and vomiting [4]. Clinical MTX intolerance often takes the form of anticipatory and associative nausea, a common side effect also observed among cancer patients [5]. A recent survey demonstrated that up to one half of pediatric JIA patients are affected by these symptoms, leading to refusal and premature discontinuation [6].

Hence, the majority of cases of MTX intolerance appear to constitute a conditioned response. However, it is possible that a subclinical toxicological effect might be the root cause of the symptom complex. Two mutations in the gene for methylene tetrahydrofolate reductase (MTHFR), C667T and A1298C, are the most common cause of toxicity in high-dose MTX therapy, as these mutations lead to delayed drug clearance [7].

The objective of this study was to investigate associations between MTHFR gene mutations and MTX intolerance in patients with JIA.

## Methods

Consecutive patients seen at the German Center for Pediatric and Adolescent Rheumatology from October 2012 until December 2014 were included in this study after written confirmation of informed consent. The study was approved by the ethics committee of the Bayerische Landesärztekammer, Munich, Germany. Inclusion criteria were 1) diagnosis of JIA [8] and 2) treatment with MTX for at least 3 months prior to inclusion. Exclusion criteria were other conditions associated with nausea and/or abdominal complaints, and concomitant medications possibly inducing nausea (excluding biologics and non-steroidal anti-inflammatory drugs). The following data were extracted from patient medical records: age, gender, body weight and height, age at diagnosis, duration of disease, methotrexate dose, route of administration, folic acid supplementation (yes/no). In addition, liver function tests (GOT/GPT) were documented (elevated/within normal range) as well as treatment with non-steroidal anti-inflammatory drugs (NSAID) and TNFα-antagonists (TNFA).

Intolerance to MTX was measured using the Methotrexate Intolerance Severity Score (MISS) questionnaire, which has previously been validated for use in JIA [6]. This questionnaire consists of four domains: abdominal pain, nausea, vomiting and behavioral symptoms, and records symptoms before (anticipatory) and after MTX administration, as well as associative symptoms (i.e., symptoms occurring when thinking about MTX). Symptoms are assessed at the time of answering the questionnaire, with no specific time frame given. Behavioral symptoms include restlessness, irritability and refusal of MTX in response to gastrointestinal symptoms. Each of the 12 items of the MISS scale is scored on a Likert scale (0 = no symptoms, 1 = mild symptoms, 2 = moderate symptoms; 3 = severe symptoms) for a maximum score of 36. As previously suggested, MTX intolerance was defined as ≥ 6 points on the MISS questionnaire, including at least one anticipatory, associative or behavioral symptom [9]. DNA was prepared from peripheral mononuclear cells using standardized protocols. Presence of the two most common mutations in the MTHFR gene (C677T and A1298C) was assayed using polymerase chain reaction, as described previously [7, 10].

Demographic and clinical data were analyzed using descriptive statistics. Associations of demographic or treatment factors with MTHFR genotypes were analyzed using univariate statistics (chi-squared test, Student’s *t*-test), where appropriate. Correlation between age and MTX dose per square meter body surface area (MTX dose/m2 BSA) was calculated using Pearson’s correlation coefficient. MTHFR mutations were analyzed directly and also grouped in categories by the amount of mutations in the two loci, using univariate non-parametric statistics (chi-squared test and Kruskal-Wallis test). For the latter, we assumed that 0, 1 and 2 mutations correspond to a locus with two alleles in which 0 and 2 are homozygous and 1 is heterozygous. Number of mutations in these two gene loci has previously been associated with MTX toxicity before [11].

## Results

Demographic data of the 196 patients included in the study as shown in Table 1. There were 90 (90/196 = 46 %) patients who were considered MTX intolerant (MISS: median 11, range 6–32). Table 2 shows univariate analysis of demographic and clinical factors on presence of MTX intolerance. Disease duration was significantly associated with occurrence of MTX intolerance. No other factors showed any significant correlation, although there were non-significant correlations with age, duration of MTX treatment and folic acid supplementation. Higher doses of MTX were non-significantly correlated with a lower frequency of MTX intolerance (*p* = 0.026); there was an inverse correlation between age and MTX dose/m^2^ BSA (*r*^2^ = 0.788, *p* < 0.001)

**Table 1:** Demographic and clinical data of study participants

Patients	(*n* = 196)
Age (median, range)	10,7 years (3,1–17,9 years)
Female gender (%)	141 (72 %)
Disease duration (median, range)	3,8 years (15,9-0,5 years)
Duration of MTX treatment (median, range)	1,2 years (13,2- 0,3 years)
Patients with MTX intolerance (%)	90 (45,9 %)
Patients on folic acid	184 (94 %)
Patients on folic acid with MTX intolerance	84 (93 %)

**Table 2:** Influence of demographic and clinical data on MTX intolerance

	MTX tolerant	MTX intolerant	*p*
Female Gender	74/106	67/90	0. 525
Age	9,67 ± 4,42	10,83 ± 3,89	0. 055
Age at diagnosis	5,53 ± 4,09	5,13 ± 4,04	0. 495
Disease duration	4,14 ± 3,54	5,70 ± 3,78	0. 003*
Duration of MTX treatment	1,83 ± 2,33	2,45 ± 2,32	0. 064
MTX dose/m^2^ BSA	12,29 ± 2,08	11,63 ± 2,00	0. 026
s.c. dosing	47/106	40/90	1. 000
Folic acid supplementation	103/106	81/90	0. 069
Elevated LFT	18/106	22/90	0. 217
Treatment with TNFA	47/106	33/90	0. 309
Treatment with NSAID	37/106	31/90	1. 000

*BSA* body surface area, *LFT* liver function tests, *TNFA* TNFα antagonist, *NSAID* non-steroidal anti-inflammatory drug

* significant after Bonferroni correction

DNA analyses were done in 168 patients to determine C677T and A1298C genotypes. Demographic and clinical factors were also analyzed on these patients alone, with similar results (Additional file 1: Tables S3 and S4). Among these, 40 % of patients were heterozygous, and 8 % homozygous for the C677T mutation of the MTHFR gene, 48 % of patients were heterozygous, and 14 % homozygous for the A1298C mutation; these allele frequencies are comparable to published data [12, 13].

**Fig. 1:**
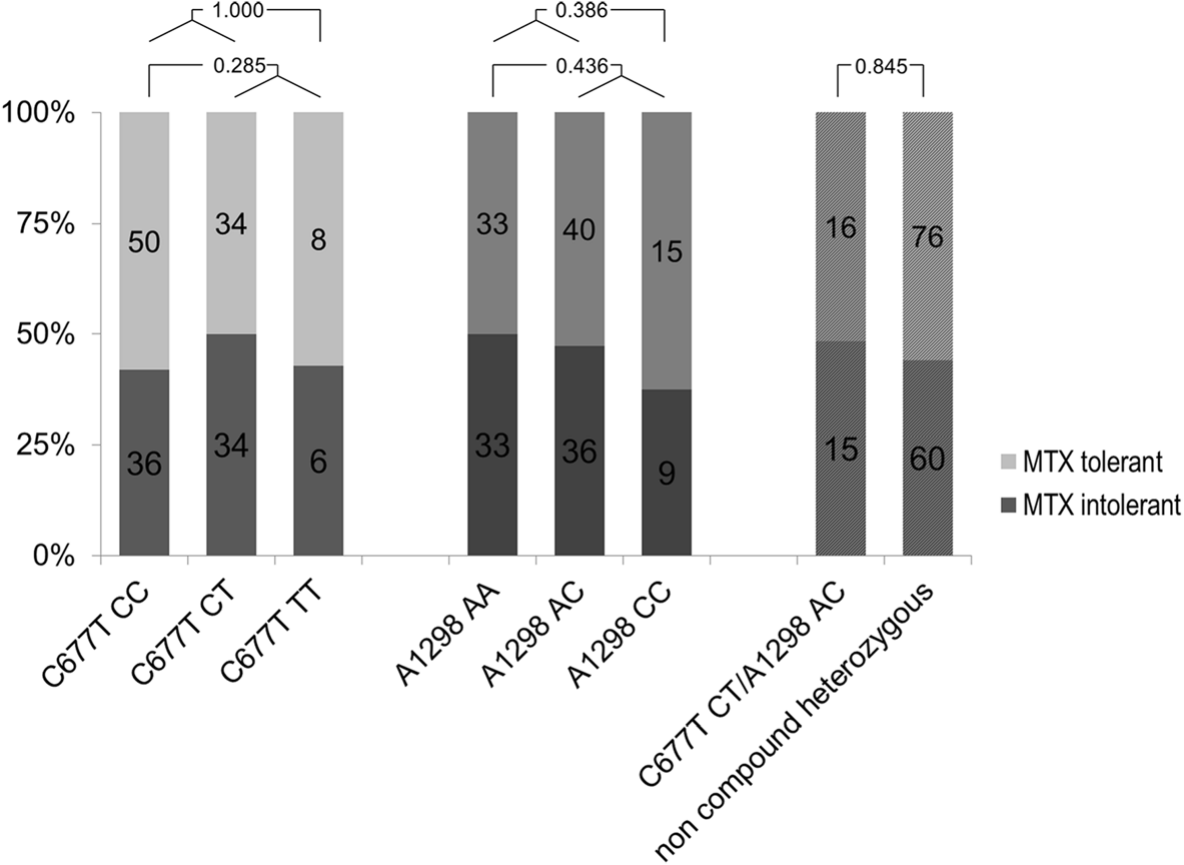
MISS scores and mutations in the C677T and A1298C loci in JIA patients on MTX. Compound and non-compound heterozygous patients are shown in the right. The numbers shown indicate p-values of the corresponding univariate analysis

**Fig. 2:**
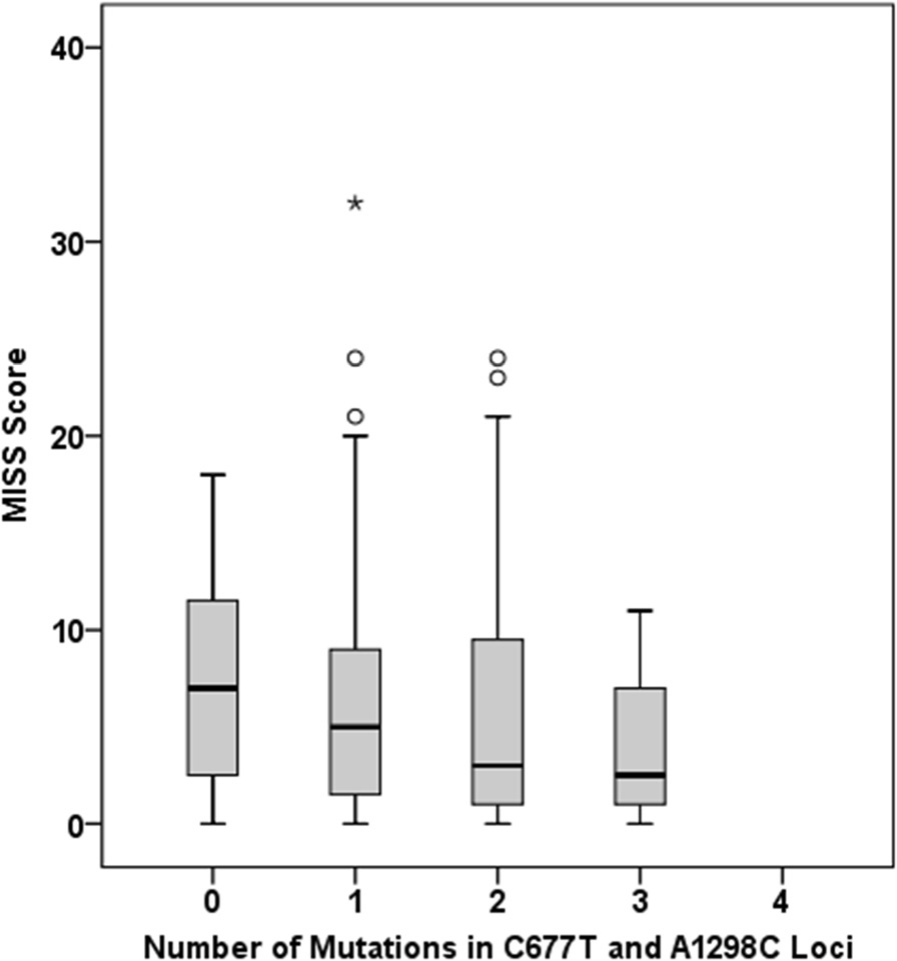
MISS scores and number of mutations in the C677T and A1298C loci in JIA patients on MTX. Note that there were no patients with mutations in all four possible loci

Compared to the homozygous wild type, MTX intolerance was not found significantly more frequently in patients with hetero- and homozygous (46 % vs. 54 %, *p* = 0.285) or homozygous (9 % vs. 8 %, *p* = 1.000) C677T mutations, nor in patients with hetero- and homozygous (64 % vs.58 %, *p* = 0.436) or homozygous (16 % vs. 11 %, *p* = 0.386) A1298C mutations. Compound heterozygous mutations for C677T and A1298C were also not significantly more frequent in patients with MTX intolerance (15 % vs. 17 %, *p* = 0.845) (Fig. 1). Analysis of the correlation between number of mutations to the MISS score did not yield a statistically significant correlation (*p* = 0.441) (Fig. 2).

## Discussion

This study demonstrates no correlation between two common mutations in the MTHFR gene and MTX intolerance in a large cohort of JIA patients. Mutations were not found more frequently in patients with MTX intolerance, nor was there any correlation between MISS score and number of mutations in the MTHFR gene. Disease duration was significantly longer in patients with MTX intolerance, and non-significant trends also showed correlation to age and duration of MTX treatment, which is not surprising. A non-significant trend toward less MTX intolerance with higher MTX doses can be explained by a clear inverse correlation between dose and age, where children at younger ages are dosed more aggressively. The observation that older age at diagnosis alone is not correlated with MTX intolerance is interesting in itself: one might expect that older children are more readily affected by a purely psychological effect.

MTX intolerance is much more frequent in younger patients than in adults [14]. It mostly occurs within the first year of treatment and worsens over time [15]. MTX intolerance in the form of anticipatory or associated nausea and vomiting has all the hallmarks of a conditioned response, where an originally neutral stimulus presented in conjunction with an unconditioned stimulus leads to a conditioned response even in the absence of the unconditioned stimulus [16]. In chemotherapy, the unconditioned stimulus clearly is drug-induced nausea; however, in low-dose MTX therapy nausea is infrequent, and cases of anticipatory nausea are possible despite lack of post-MTX nausea [6, 17]. Anticipatory nausea in other antirheumatic drugs is uncommon, despite similar rates of post-treatment nausea [18]. It is therefore reasonable to assume another, covert unconditioned stimulus as the cause of MTX intolerance.

MTX is rapidly metabolized and in low-dose therapy reaches levels of less than 5 μmol/l within 24 h [19]. Mutations in the MTHFR gene cause elevated levels of MTX in children with high-dose therapy, and in consequence, a higher and more prolonged exposure to MTX [20]. The most frequent mutations in the MTHFR gene, C677T and especially A1298 C, are associated with a higher toxicity in children and adults treated with high-dose MTX therapy [20, 21]. Influence of MTHFR on toxicity in patients treated with low-dose MTX treatment (10–15 mg/m^2^/week) is reported in some populations, but a recent meta-analysis found no clear correlation [22–24]. The C677T and A1298C mutations in the MTHFR gene also lead to higher plasma homocysteine levels, and homocysteine plasma levels have been shown to increase according to the number of mutations in an individual [11]. However, the lack of association between mutations in the MTHFR gene and MTX intolerance in this study render elevated levels of MTX and homocysteine unlikely candidates as an unconditioned stimulus in MTX intolerance.

Patients frequently receive antiemetics and a variety of home remedies in conjunction with MTX intake which might have confounded the results of this study. However, an analysis of the same patients published elsewhere showed no significant effect of these countermeasures [25]. C677T and A1298C are only two mutations in the MTX metabolism pathway, although they are by far the most common. It is possible that other genetic mutations in the pathway might be associated with MTX intolerance; a variety of mutations have been tested on a similar, but smaller population with no association [15]. The complete lack of association of the two most common mutations shown in this study renders a significant contribution to MTX intolerance by less frequent mutations unlikely. The overall strength of this study is the prospective investigation of a large population of JIA patients, allowing confidence in ruling out possible associations between these two loci and MTX intolerance.

In conclusion, mutations in the MTHFR gene, and by extension levels of MTX and homocysteine, do not play a role in the genesis of MTX intolerance. Further studies are necessary to elucidate the unconditioned stimulus underlying the conditioned response to MTX that affects a large proportion of JIA patients on this medication.

## Additional file

Additional file 1:
**Table S3.** Demographic and clinical data of study participants; patients where MTHFR testing was available. **Table S4.** Influence of demographic and clinical data on MTX intolerance; patients where MTHFR testing was available. (DOCX 35 kb)
