# Supplementary material for: Mutations in the MTHFR gene are not associated with Methotrexate intolerance in patients with juvenile idiopathic arthritis
Source: Pediatr Rheumatol Online J. 2016 Feb 29;14:11. doi: 10.1186/s12969-016-0071-y (PMC4772529; doi:10.1186/s12969-016-0071-y)
Supplement: Additional file 1: — Table S3. Demographic and clinical data of study participants; patients where MTHFR testing was available. Table S4. Influence of demographic and clinical data on MTX intolerance; patients where MTHFR testing was available. (DOCX 35 kb) [file 12969_2016_71_MOESM1_ESM.docx]

**Supplementary table 3**

**Demographic and clinical data of study participants; patients where MTHFR testing was available**

| Patients | (n=168) |
| --- | --- |
| Age (median, range) | 10,5 yrs (3,1 - 17,9 yrs) |
| Female gender (%) | 122 (72,6%) |
| Disease duration (median, range) | 3,9 yrs (15,9-0,5 yrs ) |
| Duration of MTX treatment (median, range) | 1,3 yrs (13,2 - 0,3 yrs) |
| Patients with MTX intolerance (%) | 76 (45,2%) |
| Patients on folic acid | 157 (93,5%) |
| Patients on folic acid with MTX intolerance | 68 (89,5%) |

**Supplementary table 4**

**Influence of demographic and clinical data on MTX intolerance; patients where MTHFR testing was available**

|  | MTX tolerant | MTX intolerant | P |
| --- | --- | --- | --- |
| **Female Gender** | 63/92 | 59/76 | 0,225 |
| **Age** | 9,57 ± 4,45 | 10,88 ± 3,80 | 0,044 |
| **Age at diagnosis** | 5,55 ± 4,09 | 4.89 ± 3.88 | 0,289 |
| **Disease duration** | 4,01 ± 3,48 | 5.99 ± 3,83 | **0,001*** |
| **Duration of MTX treatment** | 1,76 ± 2,14 | 2,50 ± 2,27 | 0,029 |
| **MTX dose / m^2^ BSA** | 12,28 ± 2,16 | 11,60 ± 1.94 | 0,036 |
| **s.c. dosing** | 41/92 | 32/76 | 0.757 |
| **Folic acid supplementation** | 89/92 | 68/76 | 0,068 |
| **Elevated LFT** | 16/92 | 19/76 | 0.255 |
| **Treatment with TNFA** | 44/92 | 25/76 | 0.059 |
| **Treatment with NSAID** | 32/92 | 26/76 | 1,000 |

BSA – body surface area, LFT – liver function tests, TNFA – TNFα antagonist, NSAID – non-steroidal anti-inflammatory drug. *** significant after Bonferroni correction
